# Endovascular stem cell therapy reconfigures post-stroke ER dynamics via GRP78/Atlastin/CHOP axis

**DOI:** 10.1186/s13041-026-01326-x

**Published:** 2026-06-25

**Authors:** Anita Kumari, Jyoti Yadav, Pratiksha Godse, Gautam Karmarkar, Akshada Dubey, Aditya More, Chandrima Saha, Bijoyani Ghosh, Aishika Datta, Anirban Barik, Anupom Borah, Dileep R. Yavagal, Pallab Bhattacharya

**Affiliations:** 1 Department of Pharmacology and Toxicology, National Institute of Pharmaceutical Education and Research (NIPER), Ahmedabad, Gandhinagar, Gujarat 382355 India; 2 Department of Life Science and Bioinformatics, Cellular and Molecular Neurobiology Laboratory, Assam University, Silchar, Assam India; 3 Department of Neurology and Neurosurgery, University of Miami Miller School of Medicine, Miami, FL USA

**Keywords:** Ischemic stroke, Endoplasmic reticulum dynamics, Neuroprotection, Mesenchymal stem cells, Apoptosis

## Abstract

The endoplasmic reticulum (ER) resident chaperon proteins require calcium for post-translational modifications and maintaining ER integrity. Post-stroke dysregulation of ER associated calcium homeostasis leads to altered ER dynamics and neurodegeneration. Previously, we have observed that post-stroke intra-arterial mesenchymal stem cells (IA-MSCs) administration renders neuroprotection and alleviates ER stress. Subsequently, the current study aims to investigate the role of IA-MSCs in modulating the post-stroke calcium homeostasis towards regulating ER dynamics. Male SD rats were administered with 1*10^5^ IA-MSCs at 6 h following ischemic stroke. Behaviour and motor impairment were evaluated at day 1, 7, and 14. Biochemical, histopathological, protein, and gene expression studies were also performed using cortical brain tissues. IA-MSCs administration following ischemic injury led to reduced infarct size, oxidative stress, and improved functional outcomes. It also modulated the protein and gene expressions of atlastin, reticulon, climp63 responsible for changes in the ER morphology and dynamics as evident from molecular and histological studies. Additionally, a significant decrease in the level of GRP78 and calreticulin following IA-MSCs administration, suggesting the role of IA-MSCs in maintaining calcium homeostasis. Further, we have observed that IA-MSCs administration alleviated the ER-stress induced apoptosis as evident from the reduced gene and protein expression of CHOP. Thus, the study emphasizes the therapeutic potential of IA-MSCs in ischemic stroke towards regulating the calcium-mediated ER dynamics with its future possibility as one of the adjunctive therapies for ischemic stroke.

**Supplementary Information:**

The online version contains supplementary material available at https://doi.org/10.1186/s13041-026-01326-x.

## Introduction

 Ischemic stroke occurs due to a sudden decrease in blood and oxygen supply to the brain that leads to long-term functional deficits [1]. Available FDA-approved treatments for stroke involve restoration of cerebral blood flow by thrombolysis and thrombectomy [2]. Following an ischemic stroke, excessive release of glutamate overstimulates the N-methyl-d-aspartate (NMDA) receptor and leads to pathological cellular influx of calcium ions (Ca^2+^) [3]. The calcium excitotoxicity initiates the signaling cascade of neuronal injury and apoptosis [3]. In addition to the approved therapies, Ca^2+^ channel inhibitors have been used to stabilize neuronal membranes and facilitate neuronal recovery [4]. Re-establishing the balance of Ca^2+^ levels may be crucial for achieving intracellular homeostasis to prevent neuronal death and dysfunction following stroke [5]. Endoplasmic reticulum (ER) stores and maintains cellular Ca^2+^ homeostasis, which is disrupted in post-stroke conditions due to aggravated ER stress [6]. Calreticulin is an important ER protein involved in the regulation of Ca^2+^ signaling, which is upregulated in stroke conditions and exacerbates ER stress as well as dynamics [7]. The acute stage of ER stress causes unfolded protein response (UPR); however, the prolonged stress leads to uncoupling of GRP78 and PERK complex [8, 9]. Persistent or unresolved ER stress worsens cellular damage and ultimately triggers apoptosis [10]. Climp63 is a transmembrane protein involved in ER dynamics, that function as a significant regulator of Rough Endoplasmic Reticulum (RER) sheet formation [11]. Additionally, atlastin is involved in homotypic ER fusion to produce branching tubules [12, 13]. The influence of these membrane proteins leads to alteration in Ca^2+^ levels, which further induces ER fission and damages the ER morphology [14]. Several approaches that potentially overcome the limitations of stroke therapy are currently being explored [15]. Among these, cell-based therapies have shown beneficial outcomes in preclinical and clinical contexts [16]. Mesenchymal stem cells (MSCs) therapy is the most promising due to its lower immunogenic capacity and comparatively fewer ethical issues [17]. In addition, MSCs have CXCR-4 chemokine receptors, which bind to stromal-derived factor 1 (SDF1) released in the ischemic environment, thus directing the MSCs towards the infarct site [17, 18]. Previous studies from our lab have reported that IA-MSCs can induce calcineurin-mediated neuroprotection, alter BDNF/TrkB-mediated ER-stress, apoptosis, and provide neuroprotection following stroke [17, 19–21]. Further, the influence of IA-MSCs on Ca^2+^-mediated ER dynamics is yet to be explored in the context of ischemic stroke. The current study investigates the role of IA-MSCs in altering the Ca^2+^ mediated downstream cascade to regulate ER dynamics through GRP78/Atlastin/CHOP signalling pathway.

## Materials and methods

### Chemicals and reagents

All chemicals and antibodies were procured from Abcam, Invitrogen, and Sigma-Aldrich. Experiments were carried out according to earlier reports, with minor modifications wherever required.

### Animals

Adult male Sprague-Dawley rats (6–7 weeks, weighing between 220 and 250 g) were utilized. The animals were housed for 6 days at room temperature (25 ± 0.5 °C) with relative humidity (60 ± 5%) in cages with light and dark cycles. Animals had free access to food and water throughout the experiment. Animals were divided into different groups at random, and unbiased researchers assessed the results. All the tests were performed in compliance with the Committee for Control and Supervision of Experiments on Animals (CCSEA) guidelines. The study was granted by NIPER-A Institutional Animal Ethics Committee (IAEC No. NIPERA/IAEC/2022/021; NIPERA/IAEC/2023/025).

### Experimental design

The rats were categorized into six groups: (1) Sham; (2) Stroke; (3) Stroke + PBS (S + P); (4) Stroke + MSCs (S + M); (5) Stroke + Flunarizine (S + F) and (6) Stroke + Flunarizine + MSCs (S + F + M). Total 21 animals per group were included in the experiment and used for the final analysis. In the sham group, the surgery was carried out without the filament being inserted. Animals in the stroke groups underwent transient middle cerebral artery occlusion (tMCAo) for 90 min. 1 *10^5^ IA-MSCs were administered to S + M and S + F + M and 10 mg/kg flunarizine was administered to S + F and S + F + M at 6 h post-stroke [17, 20, 22]. After 1 day and 14 days of reperfusion, the animals were euthanized under anaesthesia in accordance with the approved protocol.

Following surgery, postoperative care is essential for the animal’s long-term survival as well as for reducing pain and suffering. To help the rats recover from the hypothermia caused by the anaesthetics, they were exposed to a heat lamp for 4 h following surgery. After surgery, buprenorphine and 0.9% saline were administered to reduce dehydration and post-operative pain. To recover from anaesthesia following surgery, the animals were housed in different cages, given food pellets soaked in drinking water, and given periodic injections of 0.9% saline. The animals were monitored until their euthanasia.

### Middle cerebral artery occlusion (MCAo)

**Fig. 1:**
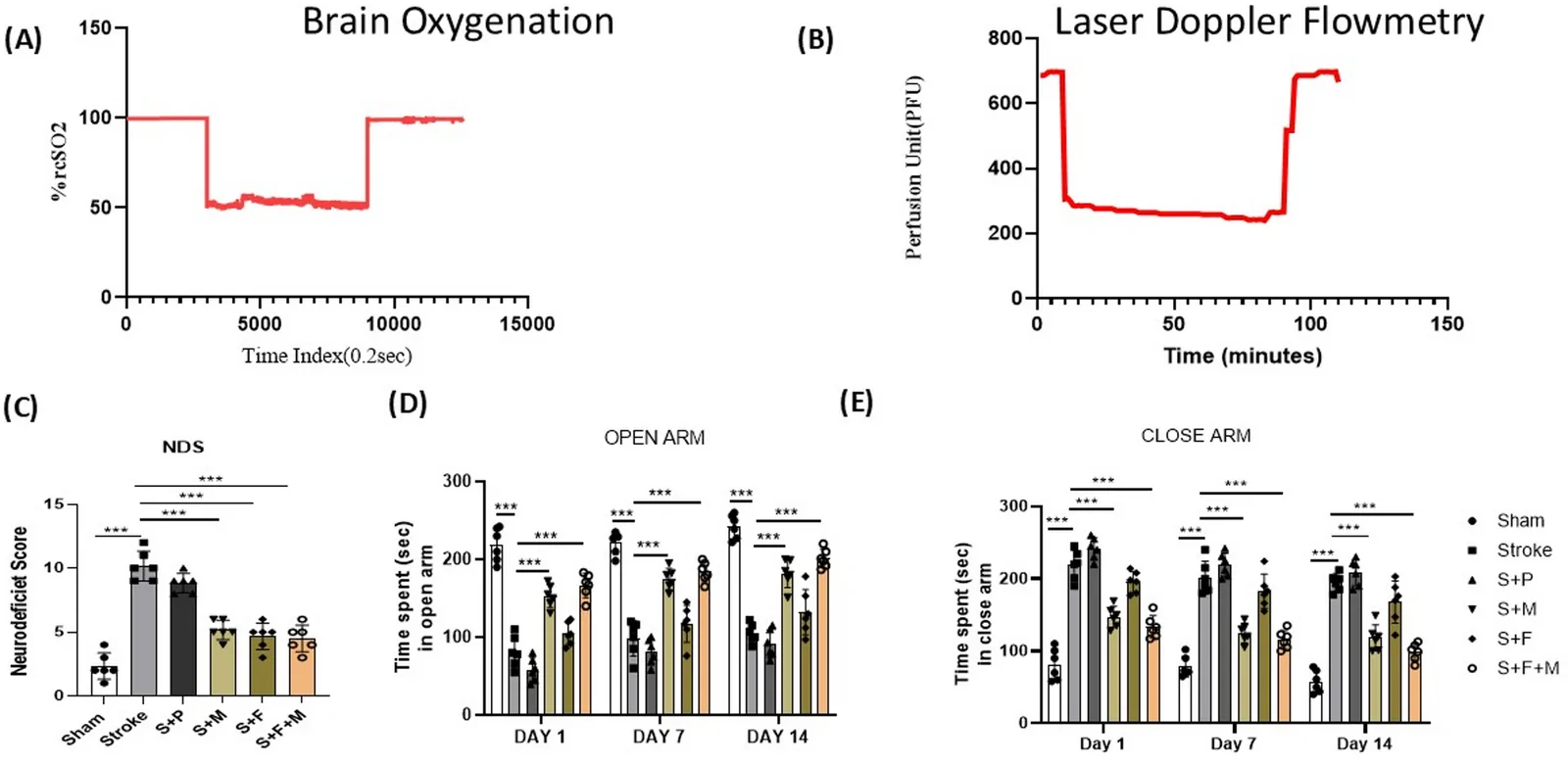
Graphical representation of** A** Brain oxygenation pre, during, and post-MCAo surgery (%rcSO_2_- Percent regional cerebral oxygen saturation).** B** Cerebral blood flow pre, during, and post-MCAo surgery measured by Laser Doppler Flowmetry (PFU-Perfusion Units). Effect of IA-MSCs on neurobehavior and motor coordination** C** Neurodeficit score,** D** time spent in open arm,** E** time spent in close arm (*n* = 6, each group). Data are expressed as mean ± SD and analyzed for statistical significance using one-way ANOVA with Tukey’s multiple-comparison test (**C**) and two- way ANOVA with Bonferroni’s post-hoc test (**D**,** E**), ***p < 0.001

According to previous studies, all surgical procedures were performed with slight changes [19, 23–25]. Blood glucose levels were monitored using One Touch^®^ glucose strips, a commercially accessible glucometer. Intubation and ventilation were performed using Harvard equipment. Gas anaesthesia was used to anaesthetize the rats (isoflurane/oxygen, 3% at induction and 1.5% during maintenance). During the procedure, arterial blood was periodically drawn from the rats’ right femoral artery, where a PE-50 catheter (BD Bioscience) was cannulated. The blood gas analyser (Radiometer) was used to monitor the blood gas parameters (pO_2_, pCO_2_, and pH) from arterial blood. Laser Doppler flowmetry (LDF) (Power Lab, AD Instruments) was used to assess cerebral blood flow (CBF) (Fig. 1A). Near-infrared spectroscopy (Moor VMS-NIRS monitor) was used to determine the regional cerebral oxygen saturation (%rcSO2) (Fig. 1B). To induce focal cerebral ischemia, animals were placed in a supine position on the surgery table and taped down using adhesive tape. Silicone coated monofilament (Doccol Corp.) was introduced through the proximal ECA into the ICA and used to occlude the middle cerebral artery (MCA) for 90-minute duration. After that, the knot in the ECA was tightened following the filament removal. Surgical sutures were used to close the midline neck incision once anterograde blood flow restoration or reperfusion was confirmed [20, 23, 26]. The animals were given the proper post-operative care and an adequate supply of analgesic.

### MSCs preparation

Rat bone marrow-derived MSCs were procured from Merck which were then cultured in aseptic expansion media. Cells were trypsinized and subjected to centrifugation at 300 g for 5 minutes after reaching confluence. 1 × 10^5^ cells were suspended in 0.5 ml of phosphate-buffered saline (PBS) and counted using the Trypan blue staining method. Thereafter, the cells were infused intra-arterially using PE-10 catheter. Rats in the IA-PBS group were administered only 0.5 ml of PBS [17–19].

### Intra-arterial administration of MSCs

6 h following MCAo, MSCs were administered into the internal carotid artery (ICA) via the IA route. A 1-ml syringe and a PE-10 polyethylene catheter (BD Bio Sciences) were used for the procedure. Heparinized physiological saline was used to fill the catheter to prevent blood coagulation and air bubbles. A small incision was made into the left external carotid artery stump to insert the tip of the PE-10 catheter. Cells were gradually injected into the distal internal carotid artery for 10 min. The remaining procedures were carried out using the techniques shown in previous reported studies [17–19].

### Behavioural assessment

All assessments were performed at days 1, 7, and 14 following reperfusions in accordance with previously published research, with a few changes. The neurodeficit score was calculated using a 12-point scale consisted of four tests: proprioceptive stimuli, visual and tactile placement test, and postural reflex test that assessed the animal’s postural response and paw placing abilities in relation to its motor, sensory, and balance deficits. The overall neurological score varied from 0 denoting no significant deficit to 12 denoting highest impairment [18, 25].

### Motor function co-ordination, cognition, and forelimb grip strength evaluation

A Rotarod test was performed to evaluate motor coordination. Rats were given 3 consecutive days of training before MCAo surgery. Each training day consists of three trials with 10-minute intervals of 5, 10, and 20 RPM. At each speed, the rod’s retention time was measured. The latency to fall from the rotarod was measured post-stroke at days 1, 7, and 14 of reperfusion [18, 20, 25]. The rats’ forelimb grip strength was measured with the help of an electronic grip strength metre. The maximum force (N) exerted by the rat’s forepaws on the sensors during its grasp was computed. The rats’ grip strength was tested on the day of the procedure. 3 readings were taken following the stroke [19]. The rat was placed in the middle of the Y labyrinth and given 5 min to explore. A camera mounted above will monitor the rat’s movements while it explores. % alternations in all three arms were measured following stroke to assess cognitive impairment [27]. The elevated plus maze was used to evaluate post-stroke anxiety. The EPM apparatus, which comprises a center region and two oppositely positioned open and closed arms. At days 1, 7, and 14 after a stroke, the duration of time spent in both open and closed arms was measured [27].

### Quantification of cerebral infarct size

Two-millimetre-thick brain sections were made using brain matrix (RWD Lifesciences) and incubated at 37 °C for 30 min in 1% 2,3,5-Triphenyltetrazolium chloride (TTC) solution prepared in 0.1 M PBS. Afterward, the sections were fixed in 4% paraformaldehyde. The infarcted brain tissue remained unstained (white), while the viable tissue was stained red. NIH Image J software was used to calculate and quantify the relative infarct area from the images [18, 19, 21].

### Histological assessment and neuronal count post-stroke

Animals were anaesthetized and perfused transcardially with normal saline at 1 and 14 days, followed by 10% neutral buffered formalin (NBF). Brain tissue was harvested and kept in formalin. Sectioning was done using Vibratome (Leica) and placed on coated slides; 30 μm sections were used for H and E staining, and 40 μm sections were used for Nissl staining. For H&E staining, tissue sections were treated with xylene for 1 min followed by incubation in Harris haematoxylin for 5 min. Subsequently, incubated in eosin Y for 30 s followed by alcohol washing. For Nissl staining, the sections were incubated with xylene for 5 min and then rehydrated for 5 min with varying alcohol gradients. Thereafter, 0.1% Cresyl violet in acetate buffer (pH 3.6) was applied to the sections for 15 min at 60 °C and rehydrated with alcohol gradient. Dibutylphthalate Polystyrene Xylene (DPX) was used to mount the stained slides. Imaging was performed using a Leica DM 750 light microscope, and neuronal count was carried out using particle analysis function in NIH ImageJ software [18, 25, 28, 29].

### Biochemical analysis

Brain was harvested at day 1 following reperfusion. 75–100 mg of ipsilateral brain tissue was weighed in 2 ml microcentrifuge tube (MCT), 10µL PMSF, and 1 ml of RIPA buffer (radioimmunoprecipitation assay) were added. Following that tissue was homogenized using micro-pestles, and amplitude 40 sonication was used for three cycles (8 s on and off) and centrifuged for 20 min at 12,000 g, 4˚C. The tissue lysate’s total protein content was measured via bicinchoninic acid assay (Pierce™ BCA Protein Assay Kit) by taking the absorbance at 562 nm [28].

In compliance with previous studies, all biochemical studies were performed with slight modifications. The degree of lipid peroxidation in the samples was assessed by measuring the levels of malondialdehyde (MDA) using the Thio barbituric acid (TBA) assay. The sample was mixed with 20% v/v glacial acetic acid, 8.1% v/v sodium dodecyl sulfate (SDS), and 0.8% w/v Thio barbituric acid, and incubated for 1 h at 95 °C in a water bath. The concentration was expressed in µM/mg of protein, and the absorbance was measured spectrophotometrically at 532 nm [19, 24]. The Griess method was used to measure the nitrite concentration in the samples and nitrosative stress. Protocol follows with incubation of Griess working reagents A (0.1%) and B (1% sulfanilamide in 5% orthophosphoric acid) with the samples in a 1:1 ratio, and they were then kept at room temperature for half an hour. After being incubated, the samples are plated on a 96-well plate and analysed using spectrophotometric analysis at 540 nm (represented as µ M/mg of protein) [23, 24]. GSH concentrations were measured by Ellman’s DTNB method, which utilises incubation of samples in 0.1 M phosphate buffer with a pH of 7.4 for 10 min at room temperature. Following that, absorbance was measured at 412 nm, and the results were expressed in µM/mg of protein [19, 24]. The catalase test was carried out using a UV spectrophotometer in first-order rate kinetics at 240 nm. The reaction depends on the interaction of the samples’ catalase level with hydrogen peroxide in phosphate buffer (pH 7.4). The results were expressed as protein U/ml/mg [19, 23, 24].

### RNA isolation and real-time polymerase chain reaction (RT-PCR)

RNA isolation was done using 1 ml of TRIzol reagent (Invitrogen). 10–20 mg of ipsilateral cortical rat brain tissue was weighed at day 1 following reperfusion, homogenized, and mixed with 300 µl of chloroform and allowed to rest at room temperature. Subsequently, samples were centrifuged at 12,000 g for 20 min at 4 °C, and the clear supernatant was collected. Then supernatant was mixed with 600 µl of cold isopropyl alcohol to precipitate RNA. The samples were stored at − 20 °C overnight and in the following day, samples were centrifuged at 12,000 g for 20 min at 4 °C to get the RNA pellet. After dissolving the pellet in Tris-EDTA buffer, it was allowed to air dry before being cleaned with 95% ethanol. A Nanodrop 2000 C spectrophotometer was used to assess the purity of the RNA. Following RNA extraction, cDNA was synthesized using the BioRad iScript cDNA synthesis kit in compliance with the manufacturer’s instructions. After that, real-time PCR was carried out using an iTaq Universal SYBR Green Supermix and a BioRad real-time PCR system. 18s, Atlastin, Reticulon, CHOP, and CKAP4 KiCqStart primers (Sigma-Aldrich) were used (primer sequence listed in Table S1) [19, 20, 30].

### Western blot analysis

30 µg of protein-containing samples were denatured for 5 min at 100 °C in a dry bath using Laemmli loading buffer, which includes 2% sodium dodecyl sulfate, 20% glycerol, 0.2% bromophenol blue, and 100 mM Tris–HCl (pH 6.8). Based on the molecular weights of the proteins, the samples and ladder were loaded onto an 8–14% SDS polyacrylamide gel. Proteins were transferred to the PVDF membrane using a semi-dry gel transfer technique (Trans-Blot^®^ Turbo™ Transfer System, BioRad) and blocked for 1 h in 5% Bovine serum albumin (BSA) at room temperature. After that, the membrane was washed with TBST and incubated overnight at 4^ο^C with primary antibodies against Atlastin (1:1000, BS-11759R), Reticulon (1:1000, MA1-06815), and S100A10 (1:1000, MA-524769), GRP-78 (1:1000, SAB5700613), Climp63 (1:1000, bs-6520R), Calreticulin (1:1000, PA5-25922), CHOP (1:1000, PA5–88116), normalized to β-actin (mouse monoclonal; 1:10,000; ab8227, Abcam). The following day, HRP-conjugated goat anti-rabbit and anti-mouse secondary antibodies (1:10,000; ab6721 and ab6789) were used for 1 h incubation at room temperature. Protein blots were seen in a Gel Doc system (Bio-Rad) using ECL (chemiluminescence substrate, Bio-Rad) substrate. Densitometry analysis was performed using NIH ImageJ software [18].

### Immunofluorescence

Immunofluorescence studies were conducted on 15 μm thick coronal brain sections at days 1 and 14. The sections were taken using a vibratome on precoated slides [29]. The sections were rehydrated using an alcohol gradient and heated at 96 °C for 10 min to unmask the antigen epitopes using sodium citrate buffer (pH 6). The tissue sections were then exposed to the appropriate blocking solution, which contained 4% BSA in TBST (for 30 min in 0.3 M glycine and 90 min in glycine-free solution). After washing the sections with phosphate-buffered saline (PBS), the sections were incubated with the primary antibodies {Atlastin (1:200, BS-11759R); Climp63 (1:200, bs-6520R); S100A10 (1:200, MA-524769); Calreticulin (1:200, PA5-25922); βIII-tubulin (TuJ1) (1:200, MA1-118); NeuN (1:200, ab104224)}, for 16 h at 4 °C. The primary antibodies were diluted 1:100 or 1:200 in PBS. In the consecutive day, the sections were washed with 1× PBST and incubated with corresponding secondary antibodies for 1 h at room temperature (without direct light exposure): Alexa Fluor^®^ 647 goat anti-rabbit (Abcam, ab150079, 1:1000), Alexa Flour^®^ 488 goat anti-rabbit (Abcam, ab150077, 1:1000), and Alexa Fluor^®^ 647 goat anti-mouse (Abcam, ab150115, 1:1000). Additionally, sections were counterstain with DAPI (4 6-diamidino-2-phenylindole) for 5 min. The treated sections were viewed using a confocal laser scanning microscope (Leica Confocal Laser Scanning Microscope; Leica TCS SP8 Microsystems) to obtain high-resolution images, and ImageJ, NIH, was used for analysis [18, 20].

### Statistical analysis

The mean value ± Standard Deviation (SD) is used to represent the data. Statistical analyses were performed using GraphPad Prism version 8 (San Diego, CA). Behavioral outcomes assessed across multiple time points (Days 1, 7, and 14), including elevated plus maze, grip strength, y maze and rotarod performance, were analyzed using two-way ANOVA with experimental groups and time as independent factors, followed by Bonferroni’s multiple-comparison post hoc test (to control false-positive results, that may arise from multiple pairwise comparisons across animal groups and different time points). Neurodeficit score, biochemical, histological, immunofluorescence, RT-PCR, and western blot datasets consisted of a single dependent variable measured across independent experimental groups and were analyzed using one-way ANOVA followed by Tukey’s multiple-comparison post hoc test (to maintain overall error rate while adjusting all pairwise comparisons).* p*-values < 0.05 were regarded as statistically significant. Using the calculator provided by “Lenth, R. V. (2006),” the sample size was determined. Java applets for sample size and power [computer software]. http://www.stat.uiowa.edu/~rlenth/power [18, 19, 23, 24].

## Results

### IA-MSCs attenuate neurodeficit, improve motor co- ordination and cognition

Post-stroke motor coordination, cognitive impairment, and neurobehavior of the rats were assessed in different groups. Animals in the stroke group had a higher neurodeficit score as compared to those in the sham group. The score was much lower in IA-MSCs, Calcium channel inhibitor (CCI), and the combination group than that of the stroke group (Fig. 1, C).

**Fig. 2:**
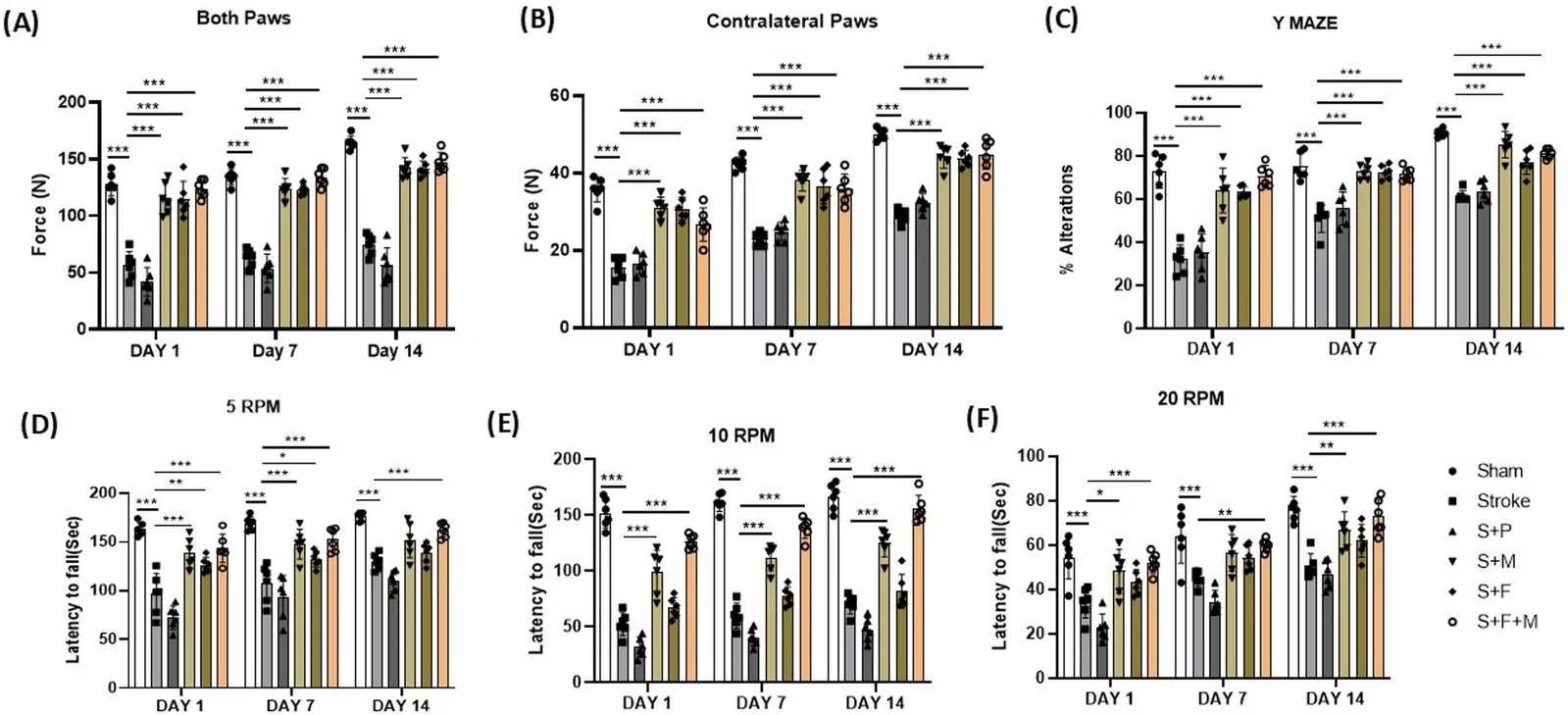
Effect of IA-MSCs on neurobehavior and motor coordination** A** Grip strength of both paws,** B** Grip strength of contralateral paws,** C** Spontaneous alterations in Y maze; Latency to fall from rotarod at** D** 5RPM,** E** 10RPM,** F** 20RPM (*n* = 6, each group). Data are expressed as mean ± SD and analyzed for statistical significance using two-way ANOVA with Bonferroni’s post-hoc test, ***p < 0.001, **p < 0.01, *p < 0.05

The rotarod test was used to evaluate the effect of IA-MSCs on motor coordination. When compared to the sham group, rats in the stroke group showed a reduced latency to fall off the rotating rod, which was improved in IA-MSCs, CCI, and combination groups at 5, 10, and 20 RPM speed on both days 1 and 14. Whereas, at day 7, no significant improvement was observed in IA-MSCs and CCI group at 20 RPM (Fig. 2, D–F). The ipsilateral and contralateral grip strength were assessed. Rats in the stroke group had significantly weaker grips than those in the sham group. However, the grip strength of both paws was significantly enhanced in IA-MSCs, CCI, and combination groups at both days 1 and 14 (Fig. 2, A–B). Y maze test was used to assess the cognitive impairment following stroke. Spontaneous alterations in the Y maze in the stroke group were less as compared to sham, IA-MSCs, CCI, and combination groups (Fig. 2, C). The elevated-plus maze was used to assess post-stroke anxiety. Rats in the Stroke group spent less time in the open arms as compared to sham, IA-MSCs, CCI, and combination groups (Fig. 1, D–E).

### IA-MSCs reduce infarct size and improve neuronal viability

**Fig. 3:**
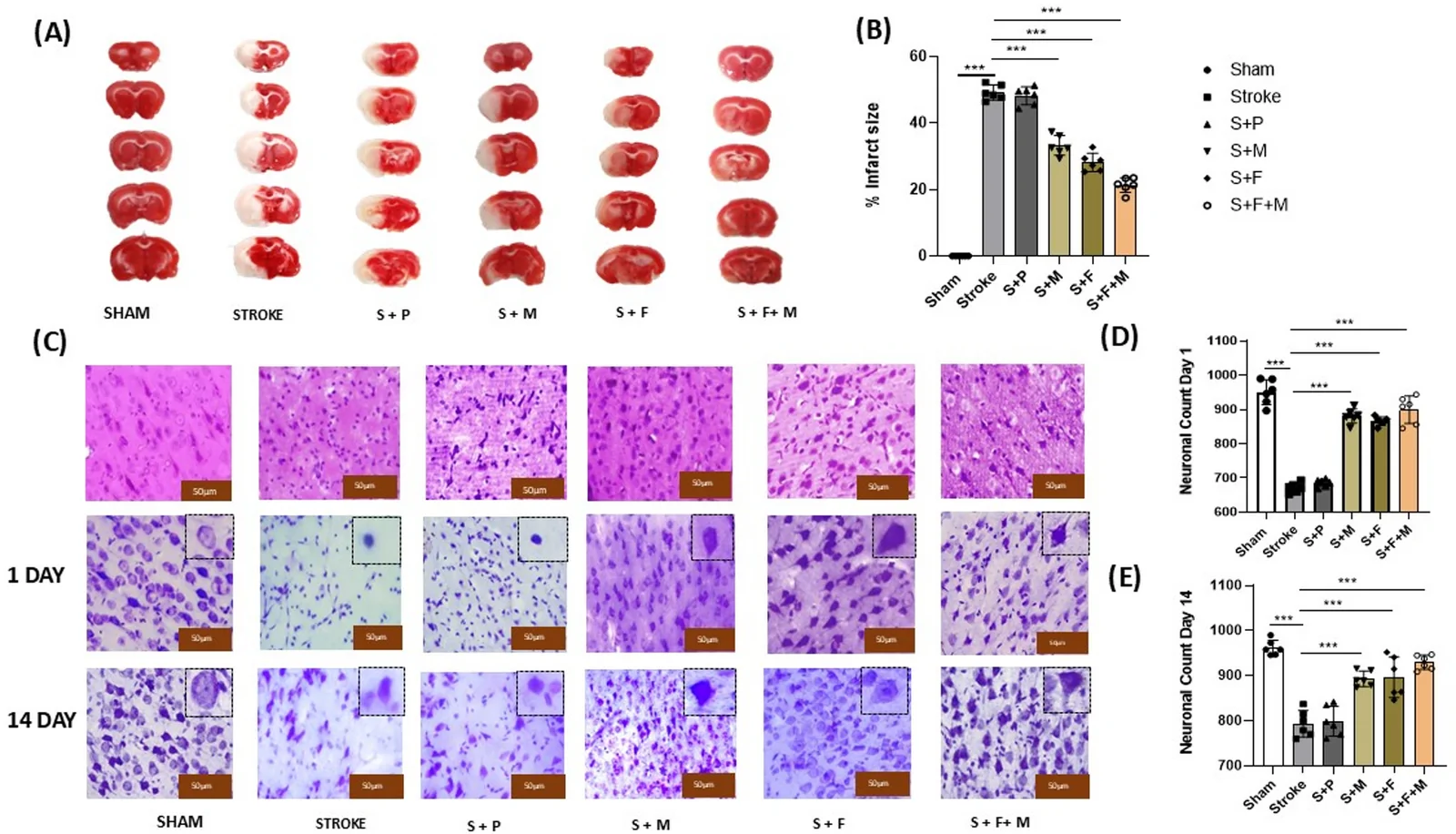
Effect of IA-MSCs on infarct area and histopathology** A** Representative TTC-stained coronal brain sections of different groups,** B** Graphs representing percent relative area infarct** C** Representative images of H&E and Nissl-stained coronal brain section (scale 50 μm),** D** Neuronal count Day 1 and** E** Day 14 (*n* = 6, each group). Data are expressed as mean ± SD and analyzed for statistical significance using one-way ANOVA with Tukey’s post hoc test, ***p < 0.001

**Fig. 4:**
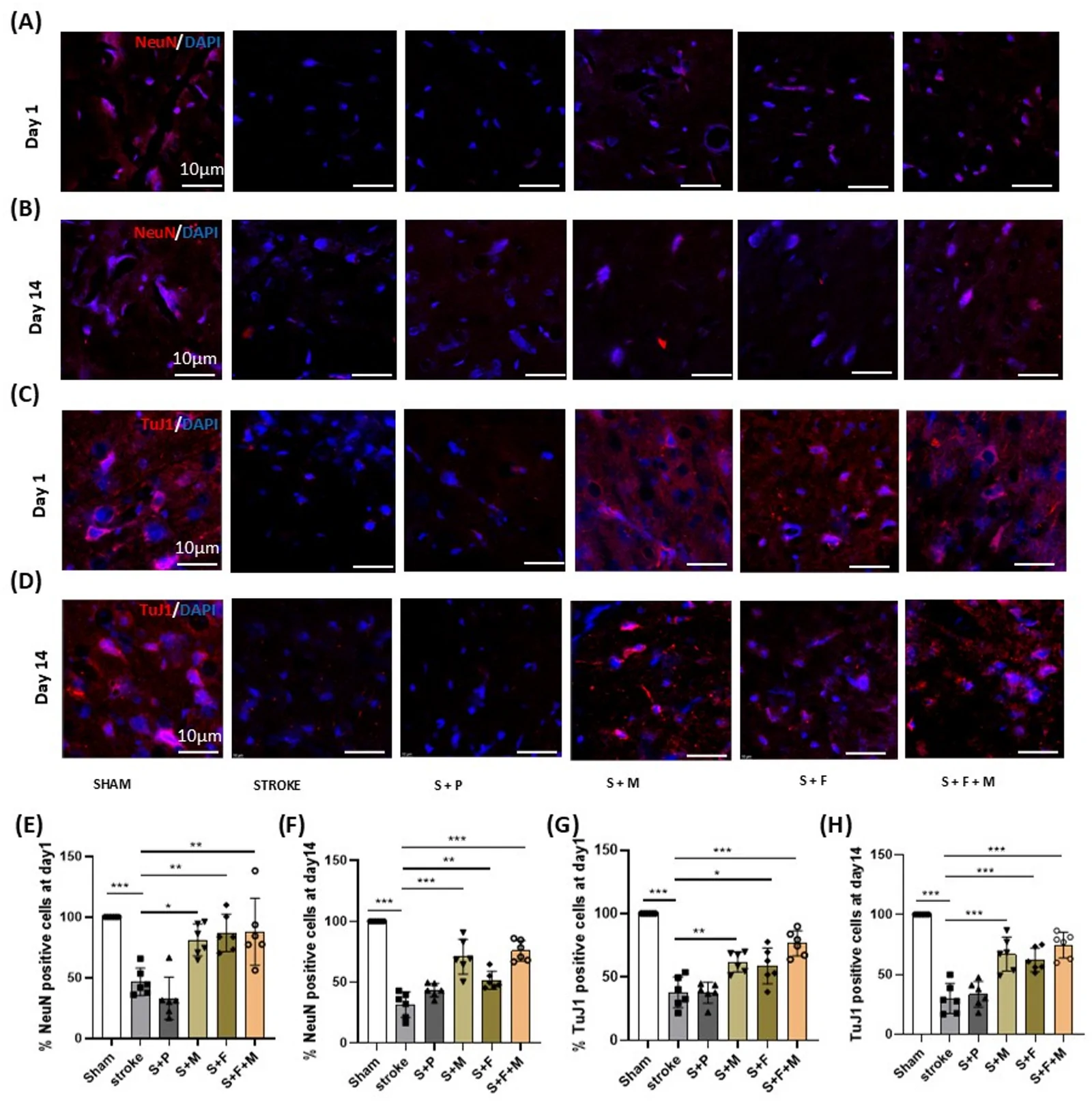
Representative immunofluorescence images showing expression of** A** NeuN at Day1,** B** NeuN at Day14,** C** TuJ1 at Day1,** D** TuJ1 at Day14, positive cells in the cortical penumbral brain region in different groups (NeuN: Red, DAPI: Blue; TuJ1: Red). Representative graph showing the** E** % NeuN positive cells at Day 1,** F** % NeuN positive cells at Day 14,** G** % TuJ1 positive cells at Day 1,** H** % TuJ1 positive cells at Day 14 (*n* = 6, each group). Data are expressed as mean ± SD and analyzed for statistical significance using one-way ANOVA with Tukey’s post hoc test, ***p < 0.001, **p < 0.01, *p < 0.05

Post-stroke infarct size was calculated using TTC staining in coronal brain sections to assess the extent of brain injury. The infarcted brain tissue remained unstained (white), while the viable tissue was stained red. Infarct size was significantly increased following stroke as compared to sham group, which was reduced in IA-MSCs, CCI and combination groups (Fig. 3, A–B). The H&E staining revealed the morphological changes in the cortical penumbral brain following stroke. Compared to the sham group, the stroke group has shown poor tissue architecture. Post stroke, the IA-MSCs group was shown to restore tissue architecture at day 1. The neuronal viability was assessed using Nissl’s staining at days 1 and 14 after reperfusion in the cortical penumbral brain following stroke. A substantial reduction in neuronal count was observed in stroke group as compared to sham group. In contrast, IA-MSCs, CCI and combination group demonstrated an increase in neuronal count as compared to the stroke group (Fig. 3, C–E). Also, neuronal identity was validated by immunofluorescence analysis of neuronal marker NeuN and TuJ1 at days 1 and 14. We observed a significant decrease in protein expression of NeuN and TuJ1 in the stroke group as compared to the sham group. Whereas IA-MSCs, CCI and combination significantly increased expression of NeuN and TuJ1, when compared to the stroke group (Fig. 4, A–H).

### IA-MSCs reduce oxidative stress and increase the activity of endogenous antioxidant enzymes following stroke

**Fig. 5:**
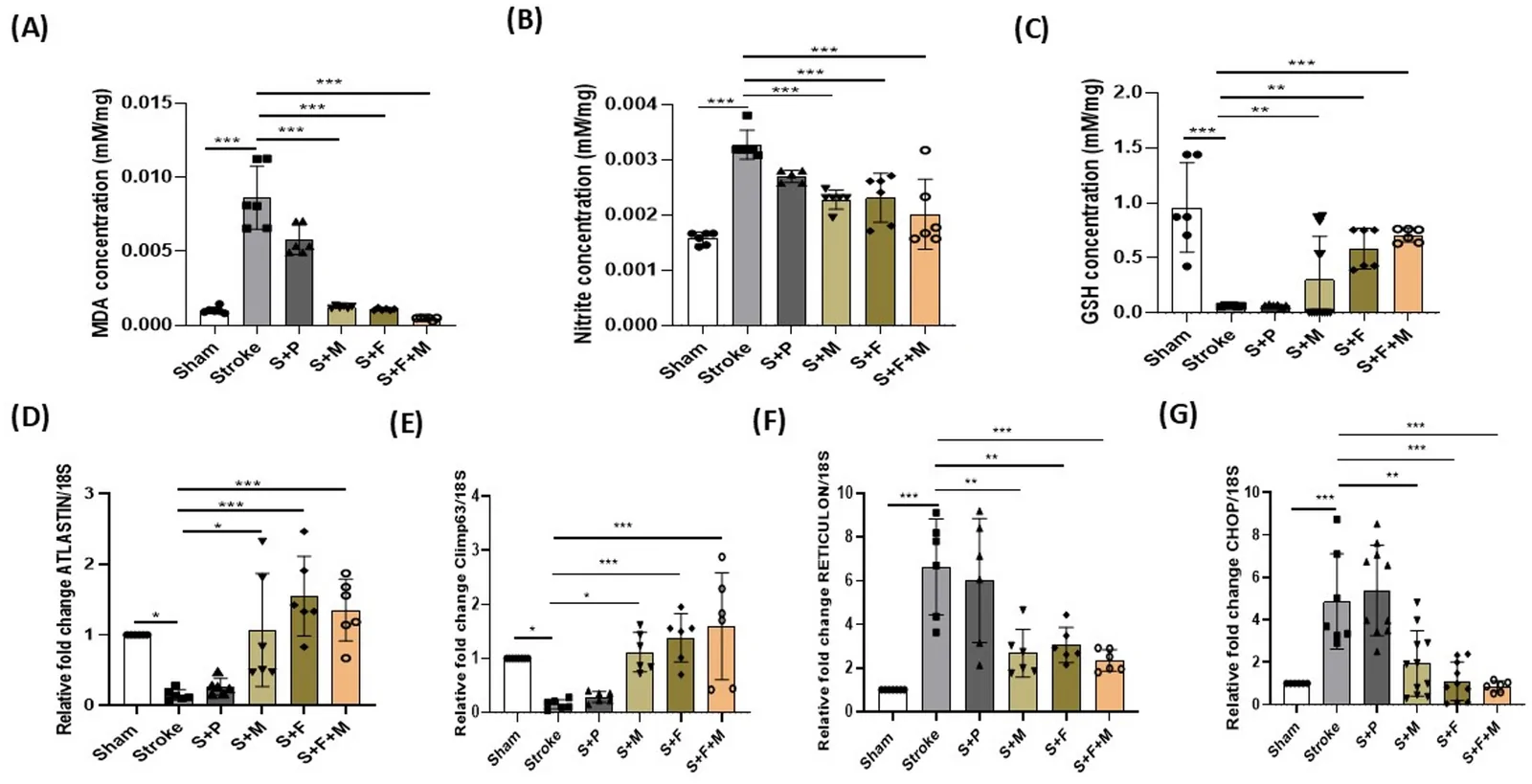
Effect of IA-MSCs on oxidative stress measured by different biochemical assays. Representative graphs of** A** GSH,** B** MDA, and** C** Nitrite. Effect of IA-MSCs on different gene expressions related to ER dynamics and apoptosis,** D** Atlastin,** E** Climp63,** F** Reticulon, and** G** CHOP (*n* = 6, each group). Data are expressed as mean ± SD and analyzed for statistical significance using one-way ANOVA with Tukey’s post hoc test, ***p < 0.001, **p < 0.01, *p < 0.05

Following stroke, enhanced intraneuronal calcium triggers the activation of oxidative stress, leading to cellular damage by enzyme dysfunction, lipid peroxidation. As evident from the biochemical assays, markedly higher levels of oxidative stress indicators (MDA and nitrite) and significantly lower levels of GSH were observed in stroke group as compared to sham group. Whereas IA-MSCs, CCI and combination group restored the MDA, nitrite, and GSH levels (Fig. 5, A–C).

### IA-MSCs regulate the mRNA expression of ER fusion, fission, and apoptosis markers

Post-stroke alteration in Ca^2+^ levels induces ER fission, damages the ER morphology and ultimately triggers apoptosis. So, based on the rtPCR results, the stroke group had decreased expression of ER fusion markers such as Atlastin and Climp63 as compared to the sham group, which were increased in IA-MSCs, CCI, and combination groups. A significantly increased expression of Reticulon in the stroke group as compared to the sham group, indicating its involvement in fragmentation and autophagic degradation of ER tubules. IA-MSCs and CCI group significantly reduced the expression of Reticulon when compared to stroke group. We also assessed the expression of the ER stress apoptotic marker (CHOP). In the stroke group, CHOP expression was increased when compared to the sham group, whereas IA-MSCs and Flunarizine administration significantly decreased CHOP expression (Fig. 5, D–G).

### IA-MSCs attenuate the stroke-induced alterations of calcium towards the regulation of endoplasmic reticulum dynamics

**Fig. 6:**
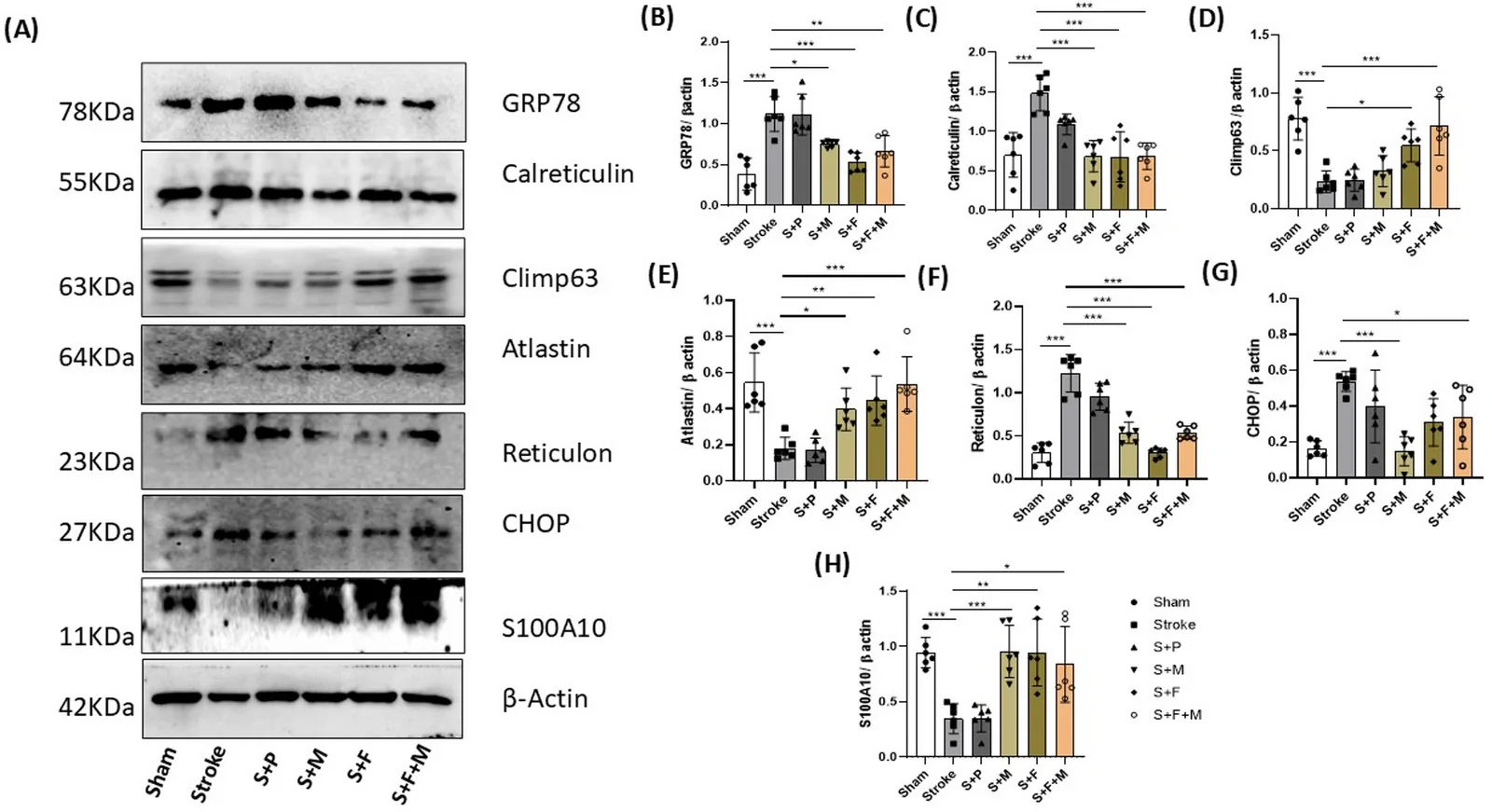
Effect of IA-MSCs on different protein expressions related to ER dynamics, calcium regulation, and apoptosis.** A** Representative immunoblots of different proteins. Representative graphs of** B** GRP78,** C** Calreticulin,** D** Climp63,** E** Atlastin,** F** Reticulon,** G** CHOP, and** H** S100A10 (*n* = 6, each group). Data are expressed as mean ± SD and analyzed for statistical significance using one-way ANOVA with Tukey’s post hoc test, ***p < 0.001, **p < 0.01, *p < 0.05

Post-stroke calcium excitotoxicity initiates the signaling cascade of neuronal injury and apoptosis. Increased protein expressions of Ca^2+^ regulation markers (Calreticulin and GRP78) were observed in the stroke group than in the sham group. These expressions were observed to be significantly lower in the IA-MSCs, CCI, and combination groups as compared to the stroke group. The Ca^2+^ binding protein S100A10 which helps in neuroprotection shows higher expression in sham, IA-MSCs, CCI and combination groups when compared to stroke group (Fig. 6, A, B, C, H).

**Fig. 7:**
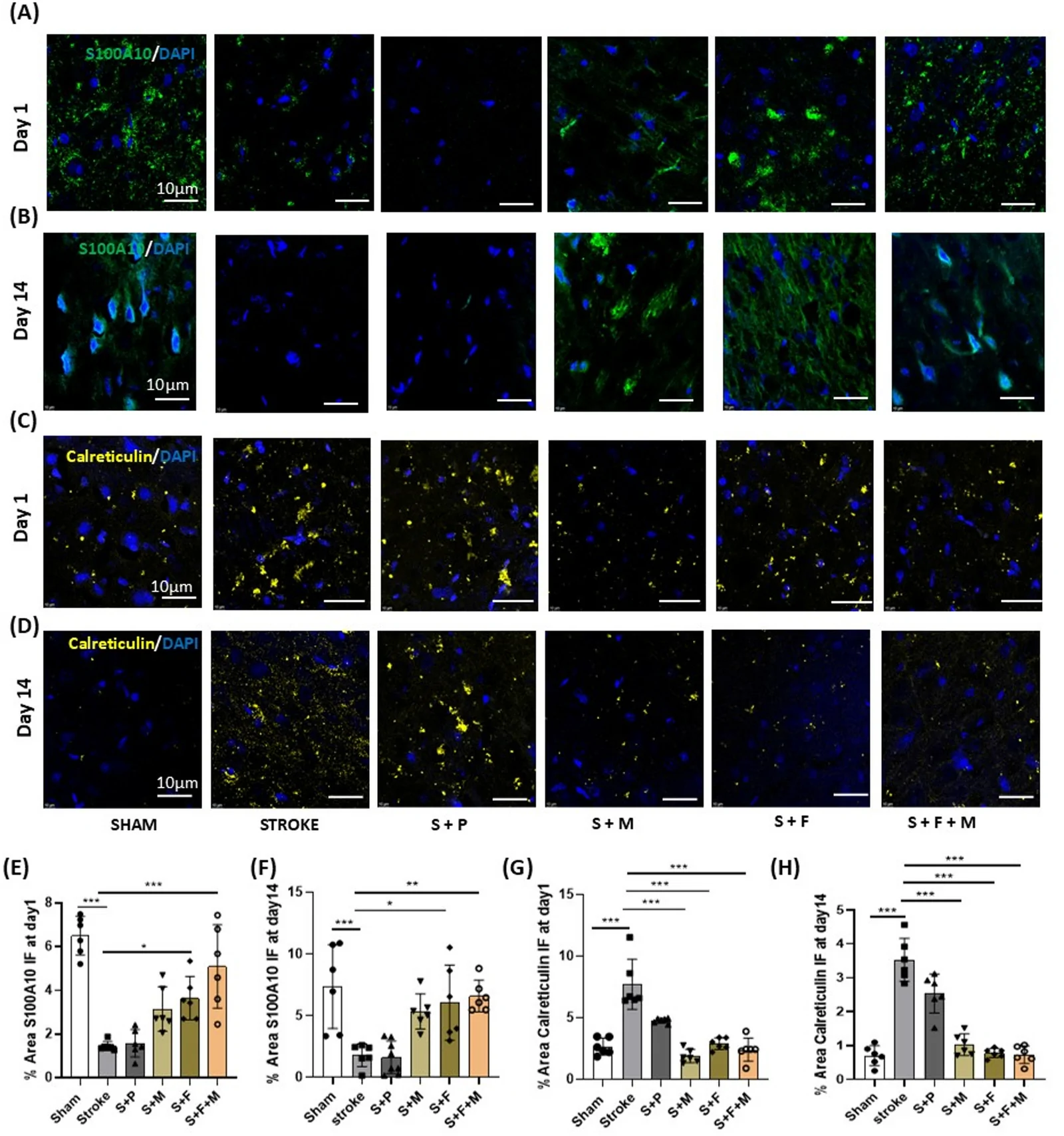
Representative immunofluorescence images showing expression of (A) S100A10 at Day1, (B) S100A10 at Day14, (C) Calreticulin at Day1, (D) Calreticulin at Day14 in the cortical penumbral brain region in different groups (S100A10: Green, DAPI: Blue; Calreticulin: Yellow). Representative graph showing the (E) % area S100A10 immunofluorescence (IF) at Day 1, (F) % area S100A10 immunofluorescence (IF) at Day 14, (G) % area Calreticulin immunofluorescence (IF) at Day 1, (H) % area Calreticulin immunofluorescence (IF) at Day 14, (n=6, each group). Data are expressed as mean ± SD and analyzed for statistical significance using one-way ANOVA with Tukey’s post hoc test, ***p < 0.001, **p<0.01, *p < 0.05

These findings were further confirmed by immunofluorescence data of Calreticulin and S100A10 at day 1 and day 14 in cortical penumbral brain region post-stroke. A significant increase in protein expression of Calreticulin was observed in the stroke group compared to the sham group. Whereas IA-MSCs, CCI and combination groups significantly decreased expression of Calreticulin, when compared to the stroke group. A significant decrease in expression of S100A10 in the stroke group was observed when compared to the sham group, which was reversed following IA-MSCs, CCI, and combination groups (Figs. 7 A–H).

### IA-MSCs regulate protein expression of ER fusion and fission markers

Post-stroke alteration in Ca^2+^ levels induces ER fission and damages the ER morphology. So, based on the western blot results, the stroke group had decreased expression of ER fusion markers such as Atlastin and Climp63 compared to the sham group. Following IA-MSCs administration, an increase in expression of Atlastin, and Climp63 was observed when compared to the stroke group. A similar trend has been observed in CCI and combination groups. A significantly increased in expression of Reticulon in stroke group was observed when compared to sham group. Whereas IA-MSCs, CCI and combination groups reduced the expression (Fig. 6, A, D, E).

**Fig. 8:**
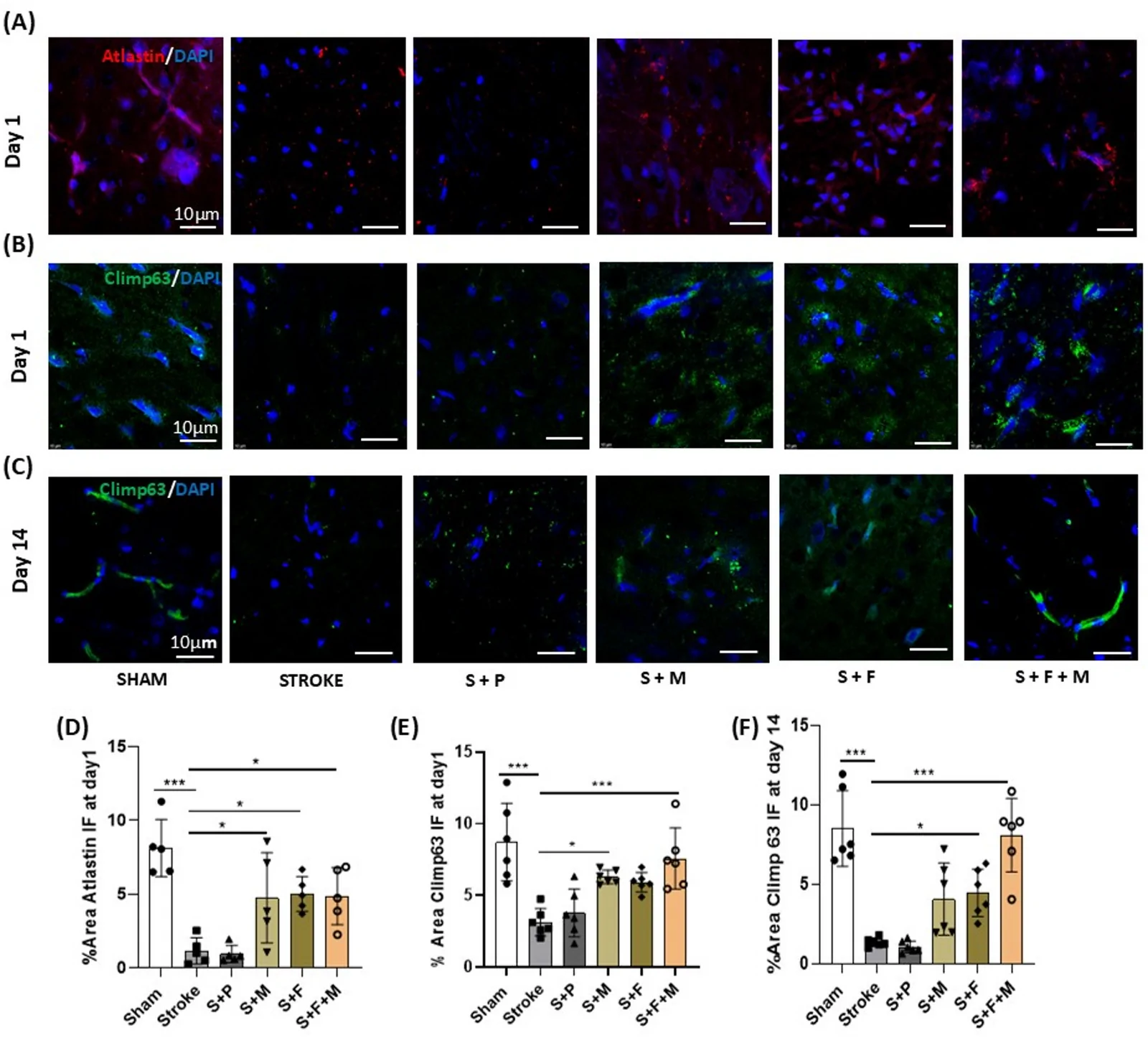
Representative immunofluorescence images showing expression of (A) Atlastin at Day1, (B) Climp63 at Day1, (C) Climp63 at Day14 in the cortical penumbral brain region in different groups (Atlastin: Red, DAPI: Blue; Climp63: Green). Representative graph showing the (D) % area Atlastin immunofluorescence (IF) at Day 1, (E) % area Climp63 immunofluorescence (IF) at Day 1, (F) % area Climp63 immunofluorescence (IF) at Day 14, (n=6, each group). Data are expressed as mean ± SD and analyzed for statistical significance using one-way ANOVA with Tukey’s post hoc test, ***p < 0.001, *p < 0.05

These western blot data were further corroborated by immunofluorescence data of Atlastin at day1 and Climp63 (ER tubule and sheet markers) at day1 and day 14 in cortical penumbral brain region following stroke. In the stroke group, Atlastin and Climp63 expression were decreased significantly when compared to sham group, indicating altered ER dynamics. IA-MSCs, CCI and combination groups substantially elevated the expression of Atlastin and Climp63 when compared to stroke group (Fig. 8A–F).

### IA-MSCs attenuate alterations of calcium-mediated apoptosis in the brain

To check the effect of IA-MSCs on apoptosis in cortical penumbral brain region, we assessed the expression of ER stress apoptotic marker (CHOP) by western blot. In the stroke group, CHOP expression was increased when compared to the sham group. In contrast, IA-MSCs, CCI, and combination groups have shown significantly decreased CHOP expression when compared to stroke group. This suggests that IA-MSCs exert a neuroprotective effect by decreasing apoptosis (Fig. 6, G).

## Discussion

Post-stroke ER stress, reactive oxygen species (ROS) generation, and disrupted neuronal ion homeostasis result in cell death [31]. During stroke, Ca^2+^ store depletes within the ER, resulting in the accumulation of misfolded proteins [6, 9]. This facilitates GRP78 (ER chaperone) dissociation from several proteins, triggering UPR activation and eventually resulting in neuronal death [9]. Post-stroke alteration in the Ca^2+^ homeostasis influences the ER membrane proteins, which further induces ER fission and alters the ER morphology [14]. Thus, to validate the role of IA-MSCs in restoring Ca^2+^ mediated altered ER dynamics following stroke, flunarizine was employed as a Ca^2+^ channel inhibitor in this study [32, 33]. Flunarizine inhibits calcium release by acting upon the ER’s inositol 1,4,5-triphosphate (IP3) and ryanodine receptors [34].

The therapeutic potential of MSCs in improving functional outcome, imparting cellular protection, along with immune regulation, tissue regeneration and neurorestoration, have been extensively studied [18, 35, 36]. Consistent with this, our lab has previously reported that IA-MSCs mitigate neurodeficit [17, 21] and alleviate ER stress by modulating the BDNF/TrkB signaling pathway following stroke [19]. However, the impact of IA-MSCs on Ca^2+^ mediated alteration of ER dynamics in the context of ischemic stroke is limitedly explored. Therefore, understanding the mechanism to restore the ER integrity and dynamics may be helpful to confer neuroprotection following ischemic stroke. In this context, the current study explores the role of IA-MSCs in modulating the Ca^2+^ mediated downstream cascade and ER dynamics through GRP78/Atlastin/CHOP signaling pathway.

In this study, we have observed that IA-MSCs treated as well as CCI groups improved neuromotor coordination and cognition in both short-term and long-term studies as compared to the stroke group. Post-stroke administration of IA-MSCs and the combination of CCI with IA-MSCs resulted in restoration of neuronal morphology and count after 1 day and 14 days. Following stroke, enhanced intraneuronal calcium triggers the activation of oxidative stress, leading to cellular damage by enzyme dysfunction, lipid peroxidation and apoptosis [37]. In our study, we observed that IA-MSCs treated groups increased antioxidant levels (glutathione) while reducing the concentrations of oxidative stressors (malondialdehyde and nitrite) following stroke. In post-stroke ER stress condition, the molecular chaperone, GRP78, regulates the unfolded protein response (UPR) that leads to aggravated ER stress [38]. In the present study, we found an increased protein expression of GRP78 post-stroke as compared to sham group. However, CCI significantly attenuated the expression of GRP78 and restored Ca^2+^ homeostasis to prevent neuronal death. S100A10 is a Ca^2+^ binding protein primarily involved in Ca^2+^ homeostasis and maintaining neuronal morphology [39]. In this study, we observed higher expression of S100A10 in the sham group as compared to the stroke group. Similarly, the CCI and combination of CCI with IA-MSCs groups have also shown a significant increase in the expression of S100A10 as compared to the stroke group. Calreticulin is an ER luminal Ca^2+^ sensor that controls Ca^2+^ dependent ER luminal events [40]. Calreticulin has been reported to be increased during ER stress following stroke [7]. We observed an increase in calreticulin expression in the stroke group as compared to the sham group. The calreticulin expression was significantly lower in the IA-MSCs treated group compared to the stroke group at day 1 and day 14 post-stroke. In CCI and combination groups, the protein expression of calreticulin was decreased as compared to stroke group. Further, this calreticulin has been reported to modulate ER morphology and the associated tubular network [41]. Atlastin, an ER fusion marker, is required for ER tubules network formation, shaping, and microtubule dynamics [11, 42]. Climp63 is another ER fusion protein involved in the formation and shaping of RER sheets [11]. Changes in Ca^2+^ levels induce reticulon-mediated ER fission, which damages the ER’s morphology [14]. However, post-stroke alterations of these proteins involved with ER dynamics are yet to be explored. Interestingly, in our study, we also observed an increase in the protein expression of atlastin and climp63 with a consequent decrease in reticulon expression in the IA-MSCs treated group as compared to the stroke group. This gives an insight into the therapeutic potential of IA-MSCs in modulating post-stroke ER dynamics. Morphology of ER networks may regulate the homeostasis of neuronal membrane proteins, which is essential when UPR is compromised [43]. It has also been reported that the elevation of reticulon protein leads to ER stress-induced apoptosis in the post-stroke condition [44, 45]. C/EBP homologous protein (CHOP), a pro-apoptotic protein, induces cell death during ER stress post-stroke [46]. Consistent with this mechanism, our study showed that significant upregulation of CHOP following stroke. IA-MSCs and CCI group significantly decreased the pro-apoptotic marker (CHOP) and mitigated the ER stress induced apoptosis.

The present findings demonstrate IA-MSC therapy confers neuroprotection following ischemic stroke by restoring calcium homeostasis and preserving ER dynamics through modulation of GRP78/Atlastin/CHOP axis. The lack of significant differences between the effect of MSCs in combination with CCI and CCI in singlet, suggests that MSC-mediated Ca²⁺ channel inhibition primarily drive post-stroke neuroprotection. Additionally, this study provides mechanistic insight into the role of stem cell therapy towards mitigating neuronal apoptosis, supporting its translational potential as an adjunctive therapy for stroke in the future.

## Supplementary Information

Below is the link to the electronic supplementary material.

Supplementary Material 1.

## Data Availability

Data is provided within the manuscript or supplementary information files.
