# Supplementary material for: Endovascular stem cell therapy reconfigures post-stroke ER dynamics via GRP78/Atlastin/CHOP axis
Source: Mol Brain. 2026 Jun 25;19:65. doi: 10.1186/s13041-026-01326-x (PMC13555993; doi:10.1186/s13041-026-01326-x)
Supplement: Supplementary file 1 [file 13041_2026_1326_MOESM1_ESM.docx]

**Table S1. Primer sequence**

| **S.No.** | **Target** | **Primer sequences** |
| --- | --- | --- |
| 1. | Atl3 (ATLASTIN) | F: GATAAGCGTTTACAGGTGAAG  R: TATTAGAGTCTGTAGCTGCTC |
| 2. | Rtn3 (RETICULON) | F: GTATAAGACACAGATTGACCAC  R: TGGCGTTTTAGTAACTGTTG |
| 3. | Ddit3 (CHOP) | F: GGAAACGAAGAGGAAGAATC  R: ATAGAACTCTGACTGGAATCTG |
| 4. | CKAP4 (Climp63) | F: GTCGAGCAGAAGGTACAG  R: CACTAATACGGTTGATCTCAC |
| 5. | 18S | F: ATCGGGGATTGCAATTATTC  R: CTCACTAAACCATCCAATCG |
